# 730. Emerging patterns of multidrug resistance Gram negative bacterial infections and its clinical outcome at a tertiary medical center

**DOI:** 10.1093/ofid/ofad500.791

**Published:** 2023-11-27

**Authors:** Shriya C Shetty, Shilpa Shenoy, Pai K B Asha, Rao M Sudhindra, Menona J Ankeeta, Avinash K Shetty, A Veena Shetty

**Affiliations:** KS Hegde Medical Academy (KSHEMA), Nitte (Deemed to be University), Deralakatte, Mangalore, India, 575018., Mangalore, Karnataka, India; KS Hegde Medical Academy (KSHEMA), Nitte (Deemed to be University), Deralakatte, Mangalore, India, 575018., Mangalore, Karnataka, India; KS Hegde Medical Academy (KSHEMA), Nitte (Deemed to be University), Deralakatte, Mangalore, India, 575018., Mangalore, Karnataka, India; KS Hegde Medical Academy (KSHEMA), Nitte (Deemed to be University), Deralakatte, Mangalore, India, 575018., Mangalore, Karnataka, India; KS Hegde Medical Academy (KSHEMA), Nitte (Deemed to be University), Deralakatte, Mangalore, India, 575018., Mangalore, Karnataka, India; Wake Forest School of Medicine and Brenner Children's Hospital,, Winston-Salem, North Carolina; KS Hegde Medical Academy (KSHEMA), Nitte (Deemed to be University), Deralakatte, Mangalore, India, 575018., Mangalore, Karnataka, India

## Abstract

**Background:**

Antimicrobial resistance (AMR) is a complex threat to global health security and universal health coverage. The continued escalation of AMR among Gram-negative bacteria (GNB) is a major concern due to the endemic presence of MDR and extremely drug-resistant (XDR) pathogens.In this regard, the present study aimed to asses the magnitude of MDR, its risk factors, and mortality in MDR/XDR - GNB.

**Methods:**

GNB isolates were retrieved from patients, identified and assessed for antibiotic resistance pattern using VITEK® 2 compact system. The extended spectrum β-lactamase (ESBL) production in 100 GNB isolates was determined by double disk synergy test and genes confirmed by PCR in retrospective cohort study (2011-2012). The carbapenemase production in 100 isolates was confirmed by modified carbapenem inactivation method in prospective cross-sectional study (2021-2022). The metallo β-lactamase (MBLs) were screened by combined disk test and carbapenemase genes detected by multiplex-PCR. Minimum inhibitory concentration (MIC) of colistin was determined by Broth Micro dilution method and clonal relatedness was evaluated by Multilocus sequence typing (MLST).

**Results:**

In retrospective study, all cases confirmed ESBL with high incidence of antibiotic

resistance in cephalosporins,chloramphenicol and ciprofloxacin. All isolates were susceptible to carbapenem. bla TEM and bla CTX-M were prevalent ESBL genes, with 7 % mortality. In prospective study, isolates were positive for carbapenemase with XDR pattern and 47% of mortality. 90% isolates were MBLs positive and 30% had at least two carbapenemase genes, the combination of bla OXA-48 and bla NDM-1 was highly prevalent. Colistin resistance was observed in 20% and 30% isolates with 8 and 4 µg/ml MICs, respectively. Further, multiplex-PCR confirmed the mcr genes and clonal relatedness was determined.

**Conclusion:**

The high prevalence rate of MDR-GNB from producing ESBLs (2011-12) to carbapenemase (2021-2022) was observed. Our results emphasize on antibiotic stewardship policies and regular surveillance programmes for monitoring AMR and screening of drug resistance genes. MLST, molecular surveillance, is valuable tool for rapid epidemiological investigation with precision in determining clonal relatedness

**Disclosures:**

**All Authors**: No reported disclosures

